# Novel, fast and efficient image-based 3D modeling method and its application in fracture risk evaluation

**DOI:** 10.3892/etm.2014.1645

**Published:** 2014-03-28

**Authors:** DAN LI, ZHITAO XIAO, GANG WANG, GUOQING ZHAO

**Affiliations:** 1 The First Teaching Hospital, Jilin University, Changchun, Jilin 130021, P.R. China; 2 Department of Engineering Mechanics, Jilin University, Changchun, Jilin 130022, P.R. China; 3 Department of Orthopedics, The Third Teaching Hospital, Jilin University, Changchun, Jilin 130033, P.R. China

**Keywords:** 3D modeling, finite element analysis, lumbar spine, computed tomography imaging, fracture risk

## Abstract

Constructing models based on computed tomography images for finite element analysis (FEA) is challenging under pathological conditions. In the present study, an innovative method was introduced that uses Siemens syngo^®^ 3D software for processing models and Mimics software for further modeling. Compared with the slice-by-slice traditional manual margin discrimination, the new 3D modeling method utilizes automatic tissue margin determination and 3D cutting using syngo software. The modeling morphologies of the two methods were similar; however, the 3D modeling method was 8–10 times faster than the traditional method, particularly in cases with osteoporosis and osteophytes. A comparative FEA study of the lumbar spines of young and elderly patients, on the basis of the models constructed by the 3D modeling method, showed peak stress elevation in the vertebrae of elderly patients. Stress distribution was homogeneous in the entire vertebrae of young individuals. By contrast, stress redistribution in the vertebrae of the elderly was concentrated in the anterior cortex of the vertebrae, which explains the high fracture risk mechanism in elderly individuals. In summary, the new 3D modeling method is highly efficient, accurate and faster than traditional methods. The method also allows reliable FEA in pathological cases with osteoporosis and osteophytes.

## Introduction

Finite element analysis (FEA) utilizes numerical analysis and minimizes variational calculus to approximate mathematical and physical problems. Turner *et al* (1) proposed the basic theory of FEA in 1956. FEA has since been refined to a high level of precision due to the decline in cost and increase in computing power resulting from rapid technological development. Belytschko *et al* (2) were the first to apply this technique to understand the mechanical behavior of the human spinal column. The application of FEA in biomechanics has accomplished considerable progress and has been widely used in orthopedics, dentistry and cerebrovascular diseases as an essential, complementary and effective alternative for *in vitro* studies. Models based on computed tomography (CT) imaging accurately visualize the geometric information of real human tissues and provide a reliable approach for the biomechanical analysis of bone tissues. Traditional model construction methods manually discern and draft the contour of the bone tissue on the basis of geometrical shape and size, using the initial scanned sample to construct a model appropriate for FEA study (3). The approach of these methods is simple and intuitive. However, discriminating and defining complex and subtle structures under pathological conditions is difficult. For example, performing 3D reconstruction in elderly patients with osteoporosis and degenerative osteophytes is challenging since similar gray and CT values discriminate the margin between the bone and soft tissues, which are ambiguous to the naked eye. Nevertheless, discriminating tissue margins in elderly patients is not difficult when the appropriate computer software is used. At the beginning of the 21st century, the development of CT imaging technology, along with FEA, has benefited from advances in computer power and CT-based modeling has a variety of new medical applications (4–14).

The present study introduces a 3D modeling method that applies 3D processing and modeling techniques. In this method, the margins of various tissues are differentiated automatically by software that analyzes data according to the intrinsic gray value of each tissue. The analysis results and the CT imaging data are then converted into the DICOM file format, which preserves all tissue density information. Assignment of Young’s modulus to non-uniform tissue, which is based on the empirical expressions of the correlations among density, CT value and Young’s modulus, becomes possible at this stage, particularly in bone tissues. The data is readily transferrable into other medical image processing software, including Mimics (Materialise, Leuven, Belgium) or Simpleware (Simpleware Ltd., Exeter, UK).

A new approach of model construction is proposed. The method implements automatic 3D tissue discrimination and incisions using the Siemens syngo^®^ 3D Workplace (Siemens, Munich, Germany) prior to transferring the data to Mimics. Converting CT images to a 3D model is fast, thus allowing more time for conducting FEA. The new model significantly improves analysis efficiency and research management. A preliminary fracture risk-evaluation study was performed in elderly individuals, according to FEA results on the basis of the new 3D modeling method.

## Materials and method

### Case selection

A total of 9,670 lumbar CT examination cases were provided by Jilin University (Changchun, China) between January 2008 and June 2011. The enrollment criterion was restricted to degenerative lumbar disease, excluding patients with lumbar fractures and tumors. This study was conducted in accordance with the Declaration of Helsinki and with approval from the Ethics Committee of Jilin University. Written informed consent was obtained from all participants.

### CT scan parameters

Patients were examined by 64-slice spiral CT (Siemens). The scan ranged between thoracic vertebra 11 and sacrum 1 using the following CT scan parameters: Tube voltage, 120 kV; effective tube current, 60 mV; and reconstruction thickness, 0.6 mm. Data were recorded in the DICOM format.

### Model construction by the conventional Mimics method

One individual in the 20–40-year-old age group was randomly selected for the preliminary step of the study. First, a model of the lumbar spine of this individual was constructed using conventional Mimics software. This case was prepared for the innovative model construction method in the next step. The average height of each vertebra (~3 cm) was divided into ~50 consecutive CT imaging slices. The conventional finite element model was constructed by discerning the margin of the bone tissue and by manually cutting the soft tissue slice-by-slice.

### Image processing via innovative 3D modeling method

Raw DICOM data of the lumbar spine of the patient underwent tissue marking, 3D cutting, home-positioning and multi-planar reconstruction using syngo software on the Siemens Workstation.

### Tissue marking

Raw DICOM data were transferred to the Siemens syngo CT Workstation. Slices of 1.0-mm sequence were dragged into the 3D application and the sagittal frame was activated in the ‘bone removal’ mode. Region selection, was the first step, where a default threshold of 175 HU in the body mode was selected to ensure that pixels with a CT value >175 HU showed blue markings. Secondly, the image was refined. The segmentation strength value at this stage was 50 and the noise tolerance was 600 HU. Refinement was conducted by recalling bone tissue that had been neglected by the software. Finally, rendering of the marking was completed and saved to ensure that a browser list of 3D applications remained on the workstation to allow for the extraction of DICOM data with processed tissue markings in the future.

### 3D incision

After tissue marking, the sagittal frame of the ‘bone display’ item was selected in the ‘bone removal’ mode. Next, the ‘Osseous Transparent metal VP’ of the visual reaction time in the type work list was selected. The 3D transparent display of the lumbar spine bone structure facilitated the 3D incision of the lumbar spine. The lumbar vertebra was incised using the 3D incision tools of the syngo software and then rotated 360° in the 3D transparent mode. This action constituted the only step for normal lumbar vertebra incision and did not necessitate a slice-by-slice drafting of the contour in Mimics. The ‘Osseous Shaded VP’ mode was used to recall bone tissue with low density, which had not been shown clearly in the ‘Osseous Transparent metal VP’ mode. The aforementioned steps allowed for high-efficiency extraction of the exact morphology of the lumbar vertebra structure.

### 3D orientation

The single incised lumbar vertebra was restored into its original sagittal orientation following selection of ‘the left to right’ item in oriental mode. The lumbar vertebra was further restored into its original 3D orientation following the selection of ‘home zoom/pan’ item in the oriental mode. Proper restoration of 3D vertebrae orientation after 3D incision was a critical step for achieving exact 3D matching of each data pixel with the raw DICOM data. The resulting orientations were then processed along the x, y and z axes.

### Multi-plane reconstruction

‘Parallel ranges’ in the setting mode was selected following a 3D incision to obtain a multi-plane reconstruction of a single lumbar vertebra. The reconstructed multi-plane horizontal slices, with 1.0 mm thickness and 1.0 mm interval, were arranged from the superior to the inferior section of the vertebra. Bone tissue data was saved in DICOM format after processing.

### Data transfer from syngo to Mimics and further processing

DICOM data of each vertebra were imported into Mimics for further processing. Only the data of one vertebral body were left in Mimics since the soft tissue and other vertebral bodies were eliminated during processing with the Siemens syngo 3D CT Workplace. All bone tissue information of the selected vertebra was obtained following the removal of the surrounding soft tissue by adjusting the threshold of the gray value to the minimum.

### Polishing and remeshing

The model was meshed and smoothed using Magics software included in the Mimics software. It was then remeshed using the split-based method. Minimum and maximum edge lengths of the tetrahedral elements were set at 0.7 and 0.9 mm, respectively.

### Congruence between the models constructed by traditional and innovative 3D modeling methods

Two models were constructed with the same raw DICOM data of a single vertebra to compare the congruence between the traditional and innovative methods. The two models were then transferred into the Mimics software using the same coordinates to form a merged image.

### Time consumption of the traditional and innovative 3D modeling methods

The 9,670 lumbar CT examinations in our institution’s database were divided into the following five age groups: 20–40, 41–50, 51–60, 61–70 and >70 years old. Five cases were selected randomly from each age group and models of the entire lumbar spine (L1–5) were constructed from each patient. Traditional and innovative methods were used, creating 10 models in total. Time consumption of each model was recorded and statistical analysis was performed using SPSS 10 (SPSS, Inc., Chicago, IL, USA). Comparisons between the two groups were performed by t-tests. P<0.05 was considered to indicate a statistically significant difference.

### Comparative FEA study of the lumbar spine under flexion and extension between young and elderly patients

The elastic modulus of the bone tissue in the vertebrae was assigned according to the corresponding gray scale in Mimics. The relationship between density (ρ) and gray scale is defined by the following formula: ρ = 1.6 × Hounsfield Unit + 47 (g/mm^3^).

Elastic moduli of the intervertebral discs were assigned with reference to the values measured by Gu *et al* (15). The anulus fibrosus in the normal vertebral disc had a water content of ~65%, Young’s modulus of 12.56 MPa and Poisson’s ratio of 0.4. The nucleus pulposus in the normal vertebrae disc had a water content of ~85%, Young’s modulus of 1.0 MPa and Poisson’s ratio of 0.49. By contrast, the anulus fibrosus in the vertebral disc with Grade IV degeneration had a water content of ~50%, Young’s modulus of 12.29 MPa and Poisson’s ratio of 0.39. The nucleus pulposus in the vertebral disc with Grade IV degeneration had a water content of ~76%, Young’s modulus of 1.66 MPa and Poisson’s ratio of 0.40. The intervertebral disc models of young (20–40 years old) and elderly (>70 years old) individuals were constructed on the basis of the corresponding Young’s modulus and Poisson’s ratio.

Upper body weight was simulated by a 400 N vertical downward force on the superior surface of L1. Flexion and extension were simulated by a 10,000 N.mm bending moment on the superior surface of L1. Peak stress and stress distribution were observed through FEA.

## Results

### Innovative 3D modeling method processed using Siemens syngo 3D software

**Figure 1:**
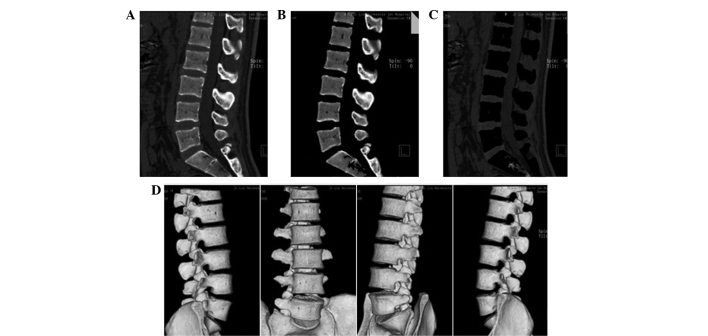
Tissue marking using Siemens syngo® software. (A) Bone and soft tissues of the lumbar spine (sagittal frame). (B) Bone tissue of the lumbar spine following tissue mark processing in the sagittal frame. (C) Soft tissue of the lumbar spine following tissue mark processing in the sagittal frame. (D) Exact single L1 structure following 3D incision. L1, lumbar 1.

Fig. 1 shows all the tissue markings with Fig. 1A showing the sagittal frame of the lumbar spine prior to tissue marking, Fig. 1B showing the sagittal frame following bone marking and Fig. 1C showing the soft tissue marking. Fig. 1D shows the 3D image of the lumbar spine following tissue marking.

### 3D incision

**Figure 2:**
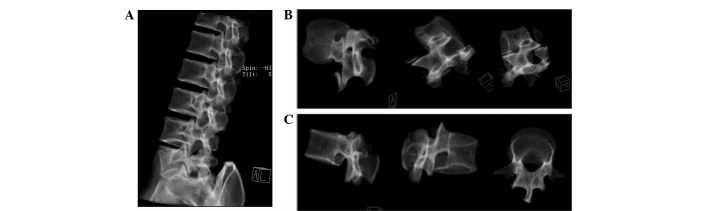
3D single L1 image extraction. (A) 3D lumbar image in Osseous Transparent metal VP mode. (B) 3D incision following 360° rotation. (C) Exact single L1 structure following 3D incision. L1, lumbar 1.

Fig. 2A demonstrates that the transparency of the bone structure significantly facilitated the 3D incision of the lumbar spine. The lumbar vertebra was then rotated 360° in a transparent mode to facilitate 3D incision (Fig. 2B) and the construction of L1 (Fig. 2C).

### Further processing with Mimics software

**Figure 3:**
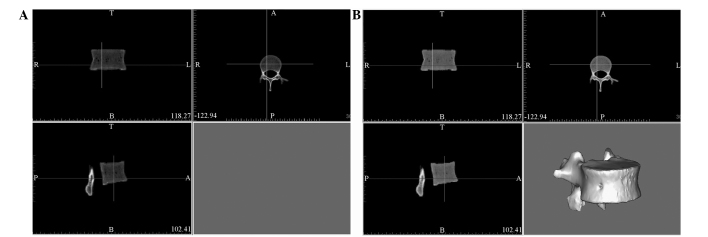
Mimics vertebral body images: (A) contour and (B) vertebral body.

DICOM data of a single vertebra were transferred into Mimics following processing by Siemens syngo 3D software (Fig. 3A). The vertebra model was constructed by further processing in Mimics (Fig. 3B). Following the removal of the surrounding soft tissue, all bone tissue information of the selected vertebra was obtained by adjusting the threshold of the gray value to the minimum. As a result, the information of the vertebral body bone tissue was completely preserved without extra information or background (Fig. 3B).

### Final model after polishing and remeshing

**Figure 4:**
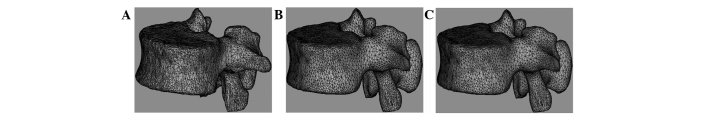
Split-based method of imaging the vertebral body. (A) Raw model prior to polishing, (B) smoothed model after polishing and (C) model after remeshing.

Fig. 4 shows the meshed, smoothed and remeshed model using the Magics software included in Mimics. Fig. 4A shows the raw model prior to polishing, Fig. 4B shows the smoothed model following polishing and Fig. 4C shows the remeshed model.

### Congruence comparison between the models constructed by traditional and innovative 3D modeling methods

**Figure 5:**
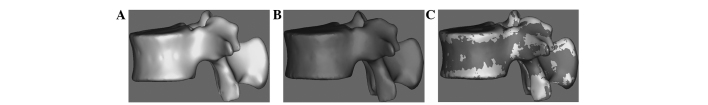
Comparison of models constructed by the (A) traditional and (B) new methods. (C) Merged image of the two vertebral models (A and B).

Models constructed by the traditional and innovative methods from the same raw DICOM data of a single vertebra are shown in Figs. 5A and 5B, respectively. The merged image of the two models (Fig. 5C) shows striking consistencies, which indicates the accuracy and reliability of the innovative method.

### Comparison of time consumption between traditional and innovative 3D modeling methods

**Figure 6:**
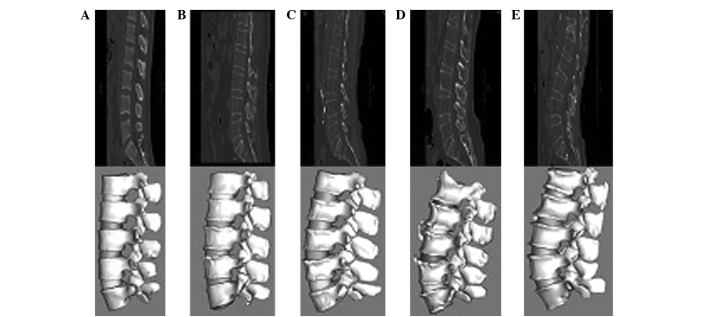
Established five lumbar models in the five age groups. A, 20–40; B, 41–50; C, 51–60; D, 61–70; and E, >70 years-old.

Fig. 6 shows the lumbar spine models constructed for five age groups by the innovative method. A significant difference was observed in time consumption of the two model construction methods. Table I shows that the time spent to produce a contour image of five age groups using the new 3D modeling method was generally shorter compared with that using the traditional method. Time consumption was longer for the elderly age groups than for younger groups, particularly when using the traditional method. Therefore, the results indicate that the difficulty of model construction increases with increasing age, and time saved by the innovative method increases as the age group increases. Constructing the model of a lumbar spine for five consecutive vertebrae (L1–5) of an elderly osteoporotic patient took ~24 h to complete using the traditional manual method, whereas ~2 h was spent to finish the same work with the new 3D modeling method. Therefore, the processing time of the innovative method was ~10-times shorter than that of the traditional method. The innovative approach markedly simplified the manual margin discrimination and slice-by-slice incision between the vertebral body and surrounding soft tissues. The new method also saved much time and greatly improved the efficiency of the analysis.

### Peak stress in the lumbar spine of young and elderly patients

**Figure 7:**
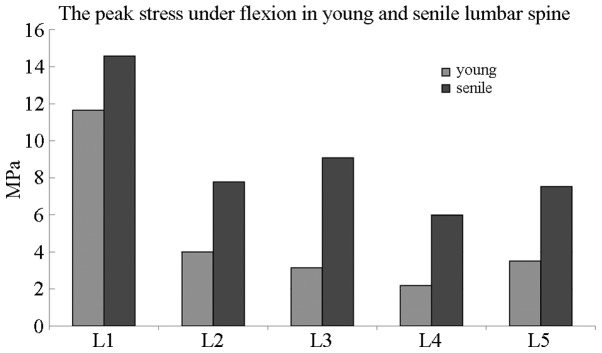
Peak stress in the lumbar spine under flexion of young and elderly patients.

Fig. 7 compares the peak stress between elderly and young individuals in the lumbar spine under flexion elevation. The most evident variations between the age groups were observed from L2 to L5, with the peak stress of elderly patients being twice as high as that of young individuals.

**Figure 8:**
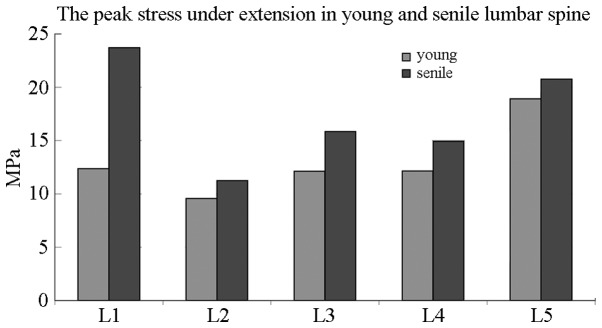
Peak stress in the lumbar spine under extension of young and elderly patients.

Fig. 8 compares the peak stress between elderly and young individuals in the lumbar spine under extension elevation. The most evident variation was observed in the L1 of elderly patients, which showed a peak stress twice as high as that of young patients.

Therefore, peak stress was elevated in each lumbar vertebra under flexion and extension in elderly people.

### Stress distribution in the median sagittal plane of L1

**Figure 9:**
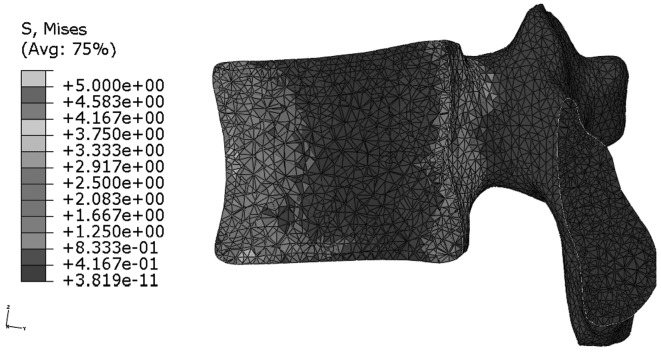
Stress distribution in the median sagittal plane of L1 under flexion in young individuals. L1, lumbar 1.

**Figure 10:**
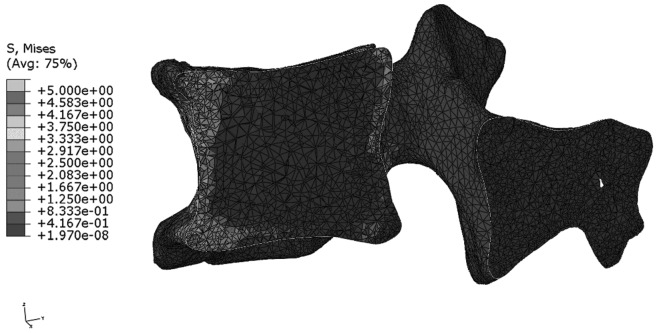
Stress distribution in the median sagittal plane of L1 under flexion in elderly individuals. L1, lumbar 1.

**Figure 11:**
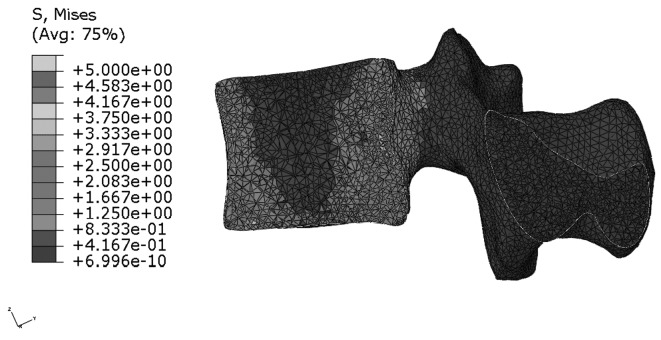
Stress distribution in the median sagittal plane of L1 under extension in young individuals. L1, lumbar 1.

**Figure 12:**
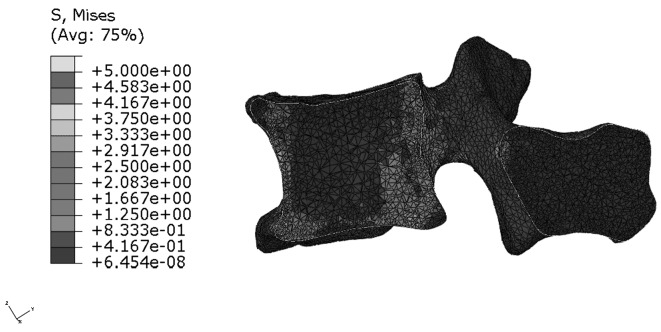
Stress distribution in the median sagittal plane of L1 under extension in elderly individuals. L1, lumbar 1.

Figs. 9 and 10 indicate that stress is evenly distributed in the entire L1 of young individuals, without stress concentration under flexion. By contrast, the stress redistribution in the L1 of older patients indicates stress-concentration in the anterior cortex under flexion. Similarly, Figs. 11 and 12 show the stress redistribution in the L1 under extension in young and elderly patients, respectively. Stress redistribution in older patients resembles stress concentration in the anterior cortex, whereas that of young individuals resembles evenly distributed stress.

## Discussion

In the last decade, significant development of biomechanics has occurred. Joint application and finite element techniques have greatly contributed to the improved understanding of biomechanical behavior in the human body, particularly the musculoskeletal system (16–18).

However, the modeling process remains complex. The current method used for constructing models from a CT scan dataset for FEA study is time-consuming and the number of samples that may be analyzed is limited (19). The traditional model construction method for processing a CT image using Mimics is meticulous and tedious work since it requires the extraction of tissues by manually drafting the contour of the vertebrae slice-by-slice (20,21). In addition, the application of FEA in objects with pathological changes has resulted in difficulties with tissue-margin discrimination for irregular pathological structures, thereby affecting the accuracy and efficiency of the traditional method. Manual discrimination of the contour of delicate osteoporotic structures and irregular osteophytes in elderly patients with typical pathological changes is ambiguous and time-consuming (22). Subsequently, miscutting of vertebral bone tissues often occurs (23). Stress distribution is greatly affected by subtle alterations in anatomical structures in pathological conditions (24). Accordingly, bias in the stage of model construction often results in unexpected deviations of the stress distribution in FEA, as the final stage of biomechanical study.

Furthermore, studies have attempted to explain stress redistribution with models based on normal anatomical configurations in pathological conditions in the early stage of FEA (3,6,7). However, these studies have failed to determine the true stress distribution in pathological conditions, particularly in cases such as osteoporosis and irregular osteophytes, which often cause more systemic errors. Moreover, margin-determination using a conventional manual model construction method is extremely time-consuming (~20 h).

In summary, the results obtained using the traditional biomechanical FEA model construction method under pathological conditions and the challenging manual determination of the contours of typical pathological changes in morphology make the traditional method tedious and prone to errors. The establishment of an accurate and efficient method of model construction has become an critical requirement for further FEA study under pathological conditions. A new 3D modeling method, that pertinently utilizes excellent multiple image post-processing with 3D processing functions of the Siemens syngo software, was developed to perform automatic tissue margin selection with high efficiency and accuracy. With the post-processing of syngo software before model construction, the soft tissue could be eliminated and the bone tissue could be extracted both accurately and effectively. This method also reduces the major limitations encountered when using the traditional construction method in creating models from pathological cases, including those encountered with elderly patients. Following multiple image post-processing to automatically select tissue margins, data are transferred from DICOM to Mimics, in which the last step of model construction for FEA study is performed. Using the new method eliminates the time-consuming slice-by-slice processing step.

A lumbar vertebra is easily rotated 360° and may be conveniently observed, incised and marked from any angle using the ‘Osseous Transparent Metal VP’ and ‘Osseous Shaded VP’ modes of the new 3D modeling method. The previously cumbersome, challenging and time-consuming task of model construction for elderly patients with osteoporosis and degenerative osteophytes has been simplified. In such cases, margin discrimination between the bone and soft tissues was formerly equivocal and misleading to the naked eye due to similarities in CT and gray values, as well as the irregular margin of the osteophytes. However, discrimination is simple when using the Siemens syngo 3D software, which removes the surrounding soft tissues to show the vertebral body alone and facilitates the final model construction in Mimics.

Comparative FEA study of the lumbar spines between young and elderly patients, on the basis of the models constructed by the new bionic modeling method, shows peak stress elevation in each lumbar vertebra of the elderly. Stress was evenly distributed throughout the vertebrae in young people, whereas stress redistribution in the vertebrae of elderly patients was concentrated in the anterior cortex, which explains the high fracture risk in the elderly. The FEA results were consistent with clinical observations, which indicated that the most common site of lumbar vertebral fracture was generally located in the anterior cortex of L1.

A fast and efficient method of CT image-based modeling of the lumbar spine for FEA is described in the present study. The model provides a 10-fold reduction in the average processing time and utilizes automatic tissue margin discrimination with Siemens syngo software in a 3D mode, instead of manual discrimination with the naked eye. In addition, 3D cutting is used instead of slice-by-slice cutting. The low speed and equivocal margin discrimination of the traditional model construction method does not allow the pathological analysis of a large number of specimens. By contrast, the new 3D modeling method achieves high efficiency and accuracy, allowing for large sample sizes and multivertebrae analysis, thereby enabling the application of significant statistical power in all future biomechanical studies. The new 3D modeling method was used to simulate the genuine mechanical situation of the lumbar spine in elderly patients, thus explaining the high fracture risk and common fracture site mechanism in this age group. Application of this method in FEA is likely to greatly contribute towards the accurate establishment of the typical characteristics of stress redistribution in any pathological condition, thereby allowing for faster and improved data accumulation for further fracture risk studies.

## Figures and Tables

**Table I:** Comparision of time consumption between traditional and new methods of model construction.

Group	Traditional method, h	SD traditional	New method, h	SD new	P-value
20–40 years	4.4	0.65	1.00	0.14	<0.001
41–50 years	13.0	1.58	1.58	0.13	<0.001
51–60 years	15.0	0.71	2.02	0.15	<0.001
61–70 years	20.4	1.14	2.12	0.08	<0.001
>70 years	24.2	1.48	2.50	0.35	<0.001
